# Recruitment and Retention of Rural-Dwelling Young Adults into a Digital Healthy Eating Intervention: Lessons Learned from a Randomized Controlled Trial of the *Veg4Me* Study

**DOI:** 10.3390/nu18111646

**Published:** 2026-05-22

**Authors:** Katherine M. Livingstone, Stephanie R. Partridge, Jonathan C. Rawstorn, Kathleen M. Dullaghan, Yuxin Zhang, Stephanie L. Godrich, Sarah A. McNaughton, Gilly A. Hendrie, Lauren C. Blekkenhorst, Ralph Maddison, John C. Mathers, Laura Alston

**Affiliations:** 1 Institute for Physical Activity and Nutrition (IPAN), School of Exercise and Nutrition Sciences, Deakin University, Geelong, VIC 3220, Australia; jonathan.rawstorn@deakin.edu.au (J.C.R.); k.dullaghan@deakin.edu.au (K.M.D.); yuxin.zhang@deakin.edu.au (Y.Z.); s.mcnaughton@uq.edu.au (S.A.M.); ralph.maddison@deakin.edu.au (R.M.); 2 Susan Wakil School of Nursing and Midwifery, Faculty of Medicine and Health, The University of Sydney, Sydney, NSW 2050, Australia; stephanie.partridge@sydney.edu.au; 3 Charles Perkins Centre, The University of Sydney, Sydney, NSW 2050, Australia; 4 School of Medical and Health Sciences, Nutrition and Health Innovation Research Institute, Edith Cowan University, Bunbury, WA 6230, Australia; s.godrich@ecu.edu.au (S.L.G.); l.blekkenhorst@ecu.edu.au (L.C.B.); 5 Health and Well-Being Centre for Research Innovation, School of Human Movement and Nutrition Sciences, University of Queensland, St Lucia, QLD 4067, Australia; 6 Health & Biosecurity, CSIRO, Adelaide, SA 5000, Australia; gilly.hendrie@csiro.au; 7 Human Nutrition & Exercise Research Centre, Centre for Healthier Lives, Population Health Sciences Institute, Newcastle University, Newcastle upon Tyne NE2 4HH, UK; john.mathers@newcastle.ac.uk; 8 Deakin Rural Health, School of Medicine, Faculty of Health, Deakin University, Warrnambool, VIC 3280, Australia; laura.alston@deakin.edu.au; 9 Research Unit, Colac Area Health, Colac, VIC 3251, Australia

**Keywords:** recruitment, retention, young adults, rural, digital, intervention

## Abstract

Background/objectives: The study aimed to identify the key methodological challenges and solutions related to recruitment and retention of rural-dwelling young adults into a randomized controlled trial that tests the feasibility of a digital healthy eating intervention (*Veg4Me*). Methods: Digital registration for a 12-week study was set up as a one-step process without researcher involvement. Participant registrations and recruitment rates were monitored daily using predetermined online preventative measures to identify fraudulent responses and to amend the digital registration process where necessary. Retention rates were monitored daily to identify any necessary amendments to the follow-up protocol. Results: During data collection, *n* = 279 fraudulent responses were identified from *n* = 536 total responses (52%). One month into recruitment, amendments were made to the registration process to reduce fraudulent responses. To address bot attacks, Qualtrics passwords and a two-factor authentication process were added to the *Veg4Me* landing page. Targeted recruitment strategies, such as unpaid social media posts, corresponded to peaks in recruitment. In the final recruitment month, a question was embedded within follow-up correspondence to encourage completion of the post-intervention survey. This resulted in an additional *n* = 8 (7%) participants completing the intervention. Conclusions: Empirical observations made in this study suggest that digital recruitment protocols without direct researcher involvement should consider multiple in-built strategies for identifying and preventing fraudulent responses. This includes a two-factor authentication process and minimizing the over-promotion of financial incentives in recruitment strategies. Recruitment strategies should consider the use of social media posts in local community groups, while the use of reminders and notifications could support retention.

## 1. Introduction

Dietary intake is a priority for improving health outcomes among rural-dwelling Australians and global populations [1,2]. There are challenges in achieving healthy diets in both urban and rural environments; however, the specific context of rural areas, including geographical isolation, must be considered in research aiming to address rural health gaps [1]. Despite this, there is limited evidence on appropriate nutrition-based interventions in rural areas [2]. Further, current evidence on healthy eating interventions in rural areas has targeted the broad adult population, with limited evidence for young adults [2,3]. The age group of 18–35 years is a key life stage for significant changes (e.g., family commitments, work/ study, and financial commitments), which are associated with risk of weight gain, sub-optimal nutrition, overweight, and obesity that track into later life [4]. Therefore, recent data suggest that the health risks faced by young adults who live in rural areas are compounded [5].

Although young adulthood is a window of opportunity for nutrition interventions to have lifelong impacts on health, historically, this population group has been difficult to recruit and retain in research studies [6,7,8]. Evidence on the reasons for these challenges and potential solutions is limited. A recent systematic review that sought to synthesize successful recruitment strategies for lifestyle interventions for 18–35-year-olds identified only 26 studies [7]. The review found that studies used both active (e.g., face-to-face) and passive (e.g., emails) ways of recruiting, but neither method was preferable to the other. The use of compensation and social media advertising was identified as a key recruitment strategy for this group. While the review did not find any rural-focused studies, using the internet and social media has been shown to be successful in recruiting young women in rural areas for other health-related research studies [8]. Similarly, research suggests that compensation can be effective for retaining young adults in web-based interventions [9], although findings are mixed [10], and evidence in rural-dwelling young adults is sparse.

Although digitally delivered interventions to support healthy eating behaviors in young adults are abundant [11,12], they remain limited in rural settings. [3] The *Veg4Me* web application (app) was among the first co-designed digital interventions that aimed to address individual and food-environment barriers to vegetable intake in young adults living in rural communities [13,14]. The advantages of digitized study processes include the streamlined online recruitment processes that can enable targeted sampling of participants from rural communities and cost-effectiveness, as well as opportunities to embed digital notifications in interventions to boost engagement and retention [15,16]. However, online research is increasingly susceptible to fraudulent responses, where individuals or automated bots provide false information to qualify for and participate in studies [17,18]. Recent estimates suggest that bot responses comprise anywhere from seven to 90 percent of the total sample size in online surveys [19,20], which can lead to false recruitment rates and negatively impact retention rates. This is particularly challenging for interventions targeting small rural communities, where the pool of eligible participants may be small, and the equity in digital health may be lacking [21]. Moreover, research on fraudulent responses in digital nutrition interventions in rural settings is extremely limited. As a result, there is a need for a better understanding of how to support researchers to overcome methodological challenges related to the recruitment and retention of rural-dwelling young adults in digitally delivered health interventions. This study aimed to identify the key methodological challenges and solutions related to recruitment and retention of rural-dwelling young adults into a web-based healthy eating intervention (the *Veg4Me* study).

## 2. Materials and Methods

### 2.1. Study Design

The *Veg4Me* trial methods have been described in detail elsewhere [14,22]. Briefly, we conducted a prospectively registered (ACTRN12623000179639), 12-week, assessor-blinded, two-arm, parallel group randomized controlled trial (RCT) from August 2023 until April 2024, consistent with the Consolidated Standards of Reporting Trials guidelines (CONSORT) [23] and the Declaration of Helsinki. All procedures involving human subjects were approved by Deakin University’s Human Ethics Advisory Group—Health (HEAG-H 06_2023). Written informed consent was obtained from all participants. The CONSORT checklist for pilot trials was used to guide reporting of the study (Supplementary Materials Table S1).

Participants randomized to the intervention group received 12 weeks’ access to the *Veg4Me* web app, which included: (1) recipes tailored to dietary and cooking preferences, (2) a geo-located local food environment map identifying rural food resources, and (3) healthy eating resources providing food literacy information. Behavior change support was available using a Qualtrics Survey Software (Qualtrics, Provo, UT, USA) goal-setting portal, and participants received weekly personalized e-newsletters.

Participants randomized to the control group received 12 weeks’ access to a non-personalized version of *Veg4Me* (browsable recipe library and a single list of rural food resources) that lacked the personalization and access to the food literacy resources, goal-setting portal, and e-newsletters.

### 2.2. Study Population

Eligible participants were aged 18–35 years (inclusive), living in the Loddon Campaspe region or Colac Otway Shire in Victoria, Australia (Modified Monash Model MM2 [regional center] to MM5 [small rural town]) [24], currently consuming <5 serves of vegetables/day, willing/able to access an internet-connected mobile device or computer and the free *Veg4Me* web app, and able to provide informed e-consent to participate. The term ‘rural’ is used throughout to refer to both regional and rural regions [25]. Participants were excluded if English was not the main language spoken at home, if they were pregnant, breastfeeding, or were currently participating in another research trial involving dietary and/or physical activity interventions. Participants with medical conditions were required to obtain clearance from their general practitioner before participating. The recruitment target was 200 participants (100 per group). With an expected dropout rate of 40%, it was expected that 120 participants (60 per group) would complete the trial [26,27,28,29,30]. This sample size was based on good practice guidelines [31].

### 2.3. Recruitment

The recruitment period ran from 7 August 2023 to 5 February 2024. Recruitment strategies used in this study are summarized in Table 1. Potential participants were recruited via local government networks, including distributing flyers with quick response codes in locations where young adults may frequent, including sporting facilities, cafes, community and neighborhood houses, and libraries. Local governments also assisted with online promotion via e-newsletters and their social media channels, which included snowballing of social media posts by community organizations. Paid advertisements on Facebook and Instagram, targeted based on age, gender, and location, were also used. A total of AUD4000 was spent over the 6-month recruitment period, with average cost per link click ranging from AUD0.78 to target males in the Loddon Campaspe Region to AUD1.19 to target females in the Colac Otway Shire. Unpaid social media posts in >20 public Facebook groups specific to the recruitment locations were identified by three researchers (KMD, KML, LA) who joined groups within each of the target communities and posted study advertisements regularly throughout the recruitment period. Two media releases (August 2023 and January 2024) resulted in four online and print articles in local media. Three in-person stalls at a local market, shopping center, and agricultural show were staggered throughout the intervention period and were run by three researchers (KMD, KML, LA). Locations were selected based on high traffic of the target age group, and offered flyers and free pens, bouncy balls, and lanyards for interested individuals. The lead researcher (KML) was invited by the local government to present at a regional conference (November 2023), where flyers were made available to local government representatives and community organizations in attendance.

### 2.4. Registration

**Figure 1:**
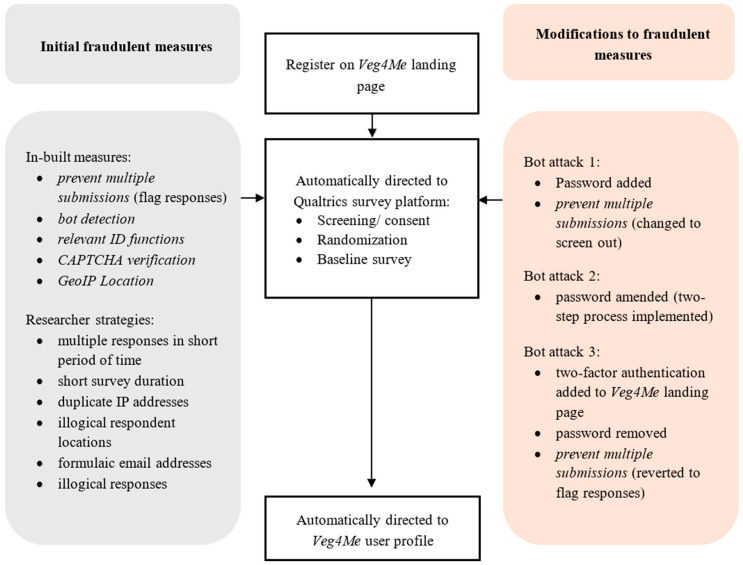
Overview of registration process and measures used to identify fraudulent responses in the *Veg4Me* study.

The *Veg4Me* registration process was initially set up as a one-step participant-facing process (Figure 1). This was intended to enable an easy process for interested individuals to register for the study. All recruitment materials included a link to the *Veg4Me* landing page, where individuals could directly access study information, including a Plain Language Statement and research team contact details. In the one-step process, individuals completed online screening questions during user website account registration. Eligible individuals were asked to indicate agreement with an online consent statement before being randomized and asked to complete the baseline survey (demographic characteristics, dietary habits, vegetable intake, digital device use). While the Qualtrics survey used in this study was developed specifically for this study, it included validated questions, details of which have been published elsewhere [14,22].

The number of participants registering for this study was monitored daily. Registrations were monitored using predetermined preventative measures (as defined below) to identify fraudulent responses and used to inform any amendments to the study protocol. This was because the one-step process used could make the study vulnerable to fraudulent responses, as it does not involve communication with a member of the research team and therefore provides no opportunity to verify eligibility. These could be either from individuals manually signing up from multiple email addresses or from bot attacks, where many accounts are created in a short space of time.

Based on the recommended criteria for defining and handling fraudulent and duplicate responses in the literature [17], in this study, several predetermined online preventative measures were implemented, the majority of which were part of available options offered within the Qualtrics survey platform. Measures used to identify these responses included *Prevent multiple submissions*, which provided a score where 1 was likely to be a duplicate, and *Bot detection*, where a score < 0.5 was likely to be a bot. The *Relevant ID functions* (*Duplicate*, *Duplicate Score*, *Fraud Score*, and *Last Start Date*) helped detect multiple responses by the same individual by looking at respondents’ metadata, where ‘True’ appears in the *Duplicate* field with the corresponding date and time the last attempt was started in the *Last Start Date* field. *Duplicate Scores* of ≥75 were indicative of duplicate responses, while *Fraud Scores* of ≥30 indicated the response was both fraudulent and likely a bot. The *Veg4Me* baseline survey also included a *CAPTCHA verification* (Completely Automated Public Turing Test to Tell Computers and Humans Apart) of “I am not a robot” to help ensure that respondents were real humans and not a program written to spam the project. Lastly, participants were automatically directed out of the survey if Qualtrics identified responses outside of Australia using *if GeoIP Location From Map Is Not#Geography.Australia*.

Potentially fraudulent responses were identified by the research team based on these in-built measures using the following handling strategies and were recorded in Microsoft Excel (Microsoft Corporation 365; Washington, DC, USA). Aligned with recent recommendations for identifying bots [20], a response was determined as being fraudulent if two researchers independently agreed it met two or more criteria: multiple responses to the registration/online survey were attempted in a short period of time (<10 min), short duration of survey completion in Qualtrics (<6 min), duplicated Internet Protocol addresses, illogical respondent locations, formulaic email addresses, atypical respondent names or illogical responses. A dual-reviewer verification process was used, where responses were first reviewed by one researcher (KMD) to determine legitimacy, then cross-checked by a second researcher (KML). The research team then discussed whether any additional strategies for counteracting fraudulent responses were necessary and implemented amendments as needed.

### 2.5. Retention

Retention in this study was based on the number of participants completing the 12-week post-intervention survey. The number of participants completing the intervention was monitored daily from 12 weeks after commencing recruitment (November 2023). The retention strategies implemented at the beginning of this study included weekly e-newsletters personalized based on the participants’ name and weekly goal (intervention group only), an AUD75 e-Gift voucher (e.g., supermarket or general retail voucher) upon completion of the post-intervention survey, and two reminder follow-up emails/SMS to encourage participants to complete the post-intervention survey. If participants started the survey but did not complete it, they received a third reminder with their personalized URL to resume their survey.

### 2.6. Data Analysis

Data were stored on password-protected Deakin University electronic servers and de-identified by assigning a unique identification number to each participant. Data analyses were conducted in Stata (Version SE 17.0; StataCorp). Data on recruitment were plotted against the timeline of recruitment strategies.

## 3. Results

### 3.1. Amendments to Study Design and Outcomes

Amendments to the study design, recruitment strategies, and/or outcomes were prioritized prior to data collection commencing, and the trial registration was updated to reflect amendments (ACTRN12623000179639). Where relevant, modifications to ethics approval were sought from Deakin University’s Human Ethics Advisory Group—Health. In August 2023, the research team redefined the feasibility outcomes. An overview of the timeline and rationale for study amendments is shown in Supplementary Materials Table S2.

Rather than the number of participants monitored, the trial registration was updated to monitor rates of recruitment and retention, in line with recommendations from a publication on recruitment strategies in young adults [26]. Recruitment rate (%) was defined as the total number of individuals randomized/total number who signed up on the *Veg4Me* website after removal of responses deemed as fraudulent and duplicate responses. Retention rate (%) was defined as the total number of participants who completed post-intervention data collection/total number who were randomized into the study. Based on previous nutrition and health interventions in young adults, and the criteria for feasibility studies [26], we considered success as a minimum 40% recruitment rate and 60% retention rate. We also estimated the total number who signed up on the *Veg4Me*/total number of adults living in the eligible regions aged 18–35, which was 0.2% (*n* = 116/*n* = 58,030). Monitoring rates of recruitment and retention throughout the study informed amendments to recruitment and retention strategies (detailed below).

Three months after recruitment commenced (November 2023), minor amendments were made to the statistical analysis plan and trial registration (Supplementary Materials Table S2). The retention rate of 60% was increased to 80% and, as a result, the recruitment target sample size reduced from 200 to 150. This is because the research team re-evaluated the estimated 40% drop out based on available literature and considered it higher than needed. This change was made after 72 people were recruited but prior to any information received from participants completing the intervention.

### 3.2. Amendments to Registration Process

During data collection, *n* = 279 fraudulent responses were identified from a total of *n* = 536 responses (52%) by the researcher (KMD). One month into recruitment (September 2023), a series of bot attacks led the research team to amend the registration process (Figure 1; Supplementary Materials Table S2). Bot attack 1 resulted in a spike in fraudulent responses, where 82% (*n* = 175) of responses during that time were fraudulent. In response to this attack, a Qualtrics password was embedded at the start of the survey in the form of a question, “What color is a carrot?”, with the answer (password) being “orange”, and the inbuilt security measure of *Prevent multiple submissions* changed from ‘flag responses’ to screening detected responses out of the survey. This briefly curtailed the bot attacks. When the bot attacks resumed the next day (bot attack 2), 100% (*n* = 46) of responses were identified as fraudulent. As a result, the researcher (KMD) changed the password and updated the text to prompt participants to contact the study email address for password access, effectively enabling a two-step registration process. This password was updated each time it was provided to a participant contacting the study email address to mitigate against exploitation by potentially fraudulent respondents. Three days later (bot attack 3), a two-factor authentication process was added to the *Veg4Me* landing page, requiring interested individuals to provide their email address, then enter an emailed code on the *Veg4Me* landing page before progressing to the Qualtrics survey platform to complete screening and, if eligible, baseline survey completion and randomization. Once this measure was enabled, the password requirement was removed from the Qualtrics survey, and the *Prevent multiple submissions* inbuilt security measure reverted back to ‘flag responses’ rather than to screen out shortly afterwards. Post-implementation of the two-factor authentication process, no further bot attacks were identified. Over a period of a week, in the month before recruitment closed, a series of fraudulent accounts were registered (fraudulent response attack). However, unlike the previous bot attacks, these accounts were created over a much more drawn-out period of time, suggesting they were manually entered rather than being automated by a bot. Following their removal, as they were created, subsequent new fraudulent registrations ceased to appear. A total of *n* = 536 accounts were registered on the *Veg4Me* landing page. After removal of individuals classified as fraudulent, failed captcha, outside Australia, and/or duplicate responses (*n* = 298), *n* = 125 individuals were eligible and provided consent to participate, and a total of *n* = 116 participants were randomized into the RCT (Supplementary Materials Figure S1).

### 3.3. Amendments to Recruitment Strategies

Four months into recruitment (December 2023), minor amendments were made to the baseline survey to collect information on how individuals had heard about the study (Supplementary Materials Table S2). This was approved as an ethics amendment. As this was a late addition, data were collected from 14 participants only. Of these, most participants reported hearing about the study from social media advertisements (43%; *n* = 6) and word of mouth (29%; *n* = 4), with others from social media groups (*n* = 1), local government communications (*n* = 1), flyers in shopfronts or local businesses (*n* = 1) or other (*n* = 1; could not remember).

**Figure 2:**
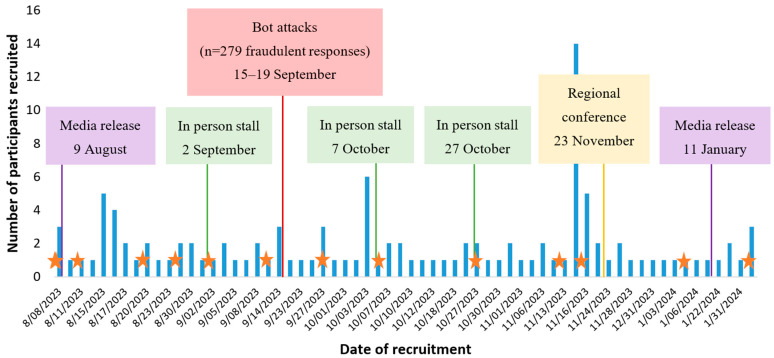
Number of recruited participants during the recruitment period from August 2023 to February 2024. Media releases, recruitment events, and bot attacks are signposted. Stars represent unpaid social media (Facebook) posts.

As shown in Figure 2, the number of participants recruited peaked following targeted recruitment activities throughout the 6-month recruitment period. Unpaid social media posts by the research team in local community groups and media releases appeared most effective for recruiting participants and so were utilized throughout the recruitment period.

### 3.4. Amendments to Retention Strategies

As outlined in Supplementary Materials Table S2, in the final month of recruitment (February 2024), a question was embedded within follow-up correspondence to participants to encourage completion of the post-intervention survey and to understand reasons for attrition: “Please select the main reason why you didn’t complete the *Veg4Me* follow-up survey:“ with options of “Forgot”, “Didn’t have time”, “Missed the invitation email”, “Not interested”, “Other (please specify)”. If the participant clicked on a response to the question within the email, it took them to Qualtrics with their response selected and followed up with “Would you still like to complete the follow-up survey?”, with “All participants who complete this survey are sent an AUD75 e-Gift card to thank them for their time.” in smaller font below this. If the participant selected “yes”, they were provided with their personalized URL to access their survey, but if they selected “No”, they were thanked for their time and were not contacted again. This correspondence was sent via email directly from the Qualtrics platform but administered manually by the research team (KMD). This question was sent to 38 participants, of whom *n* = 11 answered it. Most participants (*n* = 5, 46%) indicated that they missed the survey invitation email, with others indicating they did not have time (*n* = 3; 27%) or forgot (*n* = 3; 27%). Almost all participants (*n* = 10; 91%) who answered the question indicated that they would still like to complete the post-intervention survey, of whom 80% (*n* = 8) went on to complete the study.

## 4. Discussion

This manuscript aimed to identify the key methodological challenges and solutions related to the recruitment and retention of rural-dwelling young adults randomized into the *Veg4Me* study, a web-based healthy eating intervention. Findings from this 12-week RCT suggest that recruitment protocols for web-based interventions should consider multiple in-built strategies for identifying and preventing fraudulent responses, including a Qualtrics password and a two-factor authentication process. Moreover, in rural settings, recruitment strategies should consider the use of social media posts in local community groups, while the use of reminders and notifications could support retention.

Challenges with fraudulent responses identified in this study are consistent with previous research [17,32]. In a recent trial of UK adults recruited into an alcohol reduction app, Drink Less, 1142 participants were enrolled in the first 2 months of recruitment, of whom 76% were identified as bots during data screening [32]. Bots were identified by the research team by checking whether responses either provided a postcode that did not match the first line of the street address given or if the respondent was unknown at the phone number provided, and by the rate at which entries joined the study. However, once CAPTCHA was added, no more bots were identified [32]. In contrast, in the present study, CAPTCHA was implemented prior to the bot attack, along with many of the other Qualtrics in-built settings, but was not effective at preventing fraudulent responses from bots. The most effective strategy implemented in the present study was the addition of a two-factor authentication process, which was also recommended and implemented in the aforementioned study [32]. Similar to previous research [32], the inclusion of participant compensation is likely to have contributed to fraudulent registrations in the present study. This is supported by research suggesting that individuals and bots screen study advertisements for the inclusion of monetary incentives and symbols, such as $ [33]. While participant compensation has long been acknowledged as an effective strategy for recruitment [34], online surveys and interventions should exercise caution when promoting compensation in study recruitment materials and advertisements. Although fraudulent responses are a challenge for any online research, this is particularly problematic for research conducted in rural communities, where target communities are small, have distinct characteristics, and are often under-represented in research. Therefore, the present findings have implications for how to recruit participants in digital interventions in rural communities, with an emphasis placed on ensuring that there are in-built measures and handling strategies in place to minimize the impact of fraudulent responses. This may be particularly important for interventions that enable user-generated content and user interactions, where the impact of fraudulent behavior may go beyond receipt of participant compensation and may further exacerbate rural health inequities.

Research on recruitment strategies in rural and underserved populations has shown that best practices involve the use of technology, designed using community-based participatory research, as well as community-based recruiters [35,36,37]. This is evidenced by a recent mhealth intervention in a rural US population, where a free Facebook page that featured local involvement was the main method used for recruiting 225 participants to complete an online survey [38]. Word of mouth was the second most effective strategy, which is consistent with other research in rural communities [39,40]. In the present study, participatory research was used to design the intervention [13], and local government representatives assisted with recruitment activities. While building long-term relationships with local community actors, such as health care providers, organizations, or governments, has been shown to be an effective avenue for rural recruitment [41], substantial time investment is required to establish trusted and mutually beneficial relationships. As a result, when developing recruitment strategies for rural settings, researchers should ensure that any use of technology is viewed using a digital health equity lens, i.e., fair access to, use of, and outcomes from digital health tools across all populations [42].

Learnings for retention from this research include the need for follow-up reminders to complete surveys and mechanisms to collect data on reasons for attrition. A review of digital interventions in young adults identified that financial compensation is the most common and most effective strategy for retaining participants, with 41% of included RCTs using financial compensation, such as gift cards [26]. This is consistent with research in rural communities, where the prevalence of socio-economic disadvantage is often higher; therefore, residents may be more likely to be motivated by financial compensation [43]. The second most common strategy (21%) was contact with participants via reminders. The present study used e-Gift cards and reminders to support retention, but could have benefited from more frequent and/or notable reminders, given that participants indicated that the main reason for not completing the post-intervention survey was that they missed the email/SMS. Future research should also confirm that study emails and SMS were not screened out by junk filters (e.g., via safe lists). Nonetheless, an additional 7% of participants were successfully retained following the amendment to the last email. In this last correspondence, participants were prompted to state why they had not responded and were reminded of the compensation, which may have encouraged participants to reflect on and reconsider their survey completion. Beyond the frequency of the reminders, the format of the email prompt may have helped encourage completion of the survey, as it included an easy-to-click button linked directly to the survey. Embedding surveys within the web app has also been shown to encourage survey completion [44]. Lastly, research suggests that retention in digital interventions with rural and underserved populations also benefits from building a sense of community and connectedness [37,45], which the present study aimed to address with the inclusion of a local community food environment map.

Although our data are novel and provide valuable insights for researchers recruiting for online interventions in rural areas, more data are needed for these at-risk populations to maximize the impact of nutrition and other public health interventions. To enhance the recruitment of young adults into digital interventions in rural areas, researchers should consider including additional elements of data collection that relate specifically to barriers and enablers to online recruitment and reasons for dropout. To avoid increasing participant burden, passive data collection processes via the online platforms are recommended. More data are also needed on the best ways to reach participants. In this study, a range of recruitment strategies were used. Although a direct correlation was not measured, we observed peaks in recruitment that aligned with the timing of unpaid social media posts in local rural Facebook groups. This could also be related to the timing, including posts about completing the intervention in time for Christmas or the New Year. Future research should therefore consider the timing of the most effective strategies and whether word of mouth in smaller communities might help with recruitment. Lastly, the exclusion of pregnant/breastfeeding women may have negatively impacted rural health equity among females. Since this is a public health intervention that is not focused on weight loss, future research may also consider broadening the eligibility to include breastfeeding mothers.

Although not included here, further consideration of resource allocation and costs of different recruitment strategies would also be of benefit when maximizing research impact in resource-constrained environments (for example, the cost of addressing bot attacks and different forms of recruitment). This study shows the importance of having a pre-determined protocol for identifying fraudulent participants, and, in particular, bot attacks. Our study provides evidence that a one-step process may be less effective than a two-step process in deterring fraudulent responses from bots and is an essential consideration in recruitment. Since email and account verification are common practices in many commercial apps and web services, any perceived benefits of a more streamlined one-step registration and randomization process may not outweigh the benefits of including a verification step. Therefore, our findings reinforce the need for embedding practices that support data integrity and enable researchers to draw accurate conclusions based on survey findings. This will ensure that research resources are used appropriately and that participant compensation (e.g., a voucher) is allocated appropriately.

This study collected comprehensive data on recruitment and fraudulent participants in a large sample of rurally based young adults and is one of the first to report on recruitment and retention strategies in this population group. There are some notable limitations for this study. We excluded participants if English was not the main language spoken at home or if they were pregnant or breastfeeding, so inferences about recruitment strategies that work in these additional sub-populations cannot be drawn from this study. The data were collected from rural areas with reasonable internet coverage and may not be applicable to areas that are more remote or have internet connectivity issues. In addition, we did not undertake an economic evaluation to understand the most cost-effective recruitment strategies, as we were unable to attribute participants recruited to specific recruitment activities. A comparison of spending on paid social media advertising compared with time spent on presentations, media interviews, and unpaid posts would be useful for future research. Due to late amendments to the protocol, data on how participants had heard about the study were limited to a very small sample. As this was a key limitation of the present study, the collection of data on recruitment sources is important for inclusion in future research. These data would also provide insights into which recruitment strategies were more effective for specific subgroups within the target population. Lastly, this study defined retention based on completion of the 12-week post-intervention survey, and did not collect survey data at interim timepoints. Strategies for maintaining engagement with repeat follow-up surveys may thus require additional incentives for participants.

## 5. Conclusions

This study provides novel insights into the recruitment and retention of young rural adults to a web-based healthy eating intervention, a population who are at high risk of non-communicable diseases. Analysis of recruitment strategies from this RCT suggests that recruitment protocols for digital interventions must include strategies to mitigate against fraudulent responses, such as including two-factor authentication processes. Future communication strategies should also match the preferences of the target population, where unpaid posts in local social media groups may be effective for recruiting rural-dwelling young adults. Future research should consider collecting information on how participants heard about the study and the reasons for attrition to enhance knowledge on how best to engage rural-dwelling young adults in nutrition interventions.

## Figures and Tables

**Table 1:** Recruitment strategies used in the *Veg4Me* study.

Strategy	Details
Local government networks	Flyers with quick response codes in sporting facilities, cafes, community and neighborhood houses, and libraries. Online promotion via e-newsletters and social media channels.
Paid social media advertisements	Facebook and Instagram advertisements are targeted based on age, gender, and location.
Unpaid social media posts	Posts in public Facebook groups specific to the recruitment locations.
Media releases	Online and print articles in local media.
In-person stalls	Stalls at local markets, shopping centers, and agricultural shows, with flyers, free pens, bouncy balls, and lanyards.

## Data Availability

The datasets used and/or analyzed during the current study are available from the corresponding author on reasonable request.

## Supplementary Materials

The following supporting information can be downloaded at: https://www.mdpi.com/article/10.3390/nu18111646/s1, Figure S1: Participant flow diagram in the *Veg4Me* study; Table S1: CONSORT checklist of information to include when reporting a pilot trial; Table S2; Timeline of study amendments, rationale and number of legitimate and fraudulent participants recruited.

## Abbreviations

App	Application
CAPTCHA	Completely Automated Public Turing Test to Tell Computers and Humans Apart
RCT	Randomized Controlled Trial
