# Supplementary material for: Recruitment and Retention of Rural-Dwelling Young Adults into a Digital Healthy Eating Intervention: Lessons Learned from a Randomized Controlled Trial of the Veg4Me Study
Source: Nutrients. 2026 May 22;18(11):1646. doi: 10.3390/nu18111646 (PMC13258308; doi:10.3390/nu18111646)
Supplement: Supplementary file 1 [file nutrients-18-01646-s001.zip › Table S2.pdf]

Table S2. Timeline of study amendments, rationale and number of legitimate and fraudulent participants recruited

Time point	Amendment	Rationale	Participants recruited N (%)[1]	
			Legitimate	Fraudulent[2]
August 2023	Feasibility outcomes redefined[3]	Alignment with recommendations for recruitment strategies in young adults	–	–
15 September 2023	Password added and *prevent multiple submissions* changed from 'flag responses' to screening out of the survey	Bot attack 1	38 (17.8)	175 (82.2)
16 September 2023	Password amended and two-step process implemented	Bot attack 2	0 (0.0)	46 (100)
19 September 2023	Two-factor authentication added to *Veg4Me* landing page and password removed	Bot attack 3	1 (100)	0 (0.0)
1 October 2023	*prevent multiple submissions* changed back to 'flag responses' instead of screening out	Participant contact after making an error but not being able to re-access registration	7 (100)	0 (0)
November 2023	Statistical analysis plan updated increasing retention rate from 60% to 80%, subsequently decreasing target sample size from 200 to 150	Available literature suggested previously estimated 40% drop out was higher than warranted	–	–
8 December 2023	Question added to baseline survey	Ascertain how individuals had heard about the study	–	–
9–14 January 2024	–	Fraudulent response attack[4]	64 (67.3)	31 (32.7)
February 2024	Embedded question added within email to participants yet to complete post-intervention survey	Encourage completion of post-intervention survey and to understand reasons for attrition	7 (100)	0 (0)

1, Values represent the number (%) of legitimate and fraudulent participants recruited between each timepoint/protocol amendment

2, Discrepancies exist between participant numbers reported against amendment dates and overall recruitment numbers due to differences arising from attempted (fraudulent) registrations bypassing the *Veg4Me* landing page directly to the Qualtrics survey
3, Recruitment commenced 7 August 2023
4, A series of fraudulent accounts were registered during this timeframe but appeared to have been created manually rather than being automated, thereby not requiring any amendments to the study protocol
