# Supplementary figures and images for: Recruitment and Retention of Rural-Dwelling Young Adults into a Digital Healthy Eating Intervention: Lessons Learned from a Randomized Controlled Trial of the Veg4Me Study
Source: Nutrients. 2026 May 22;18(11):1646. doi: 10.3390/nu18111646 (PMC13258308; doi:10.3390/nu18111646)

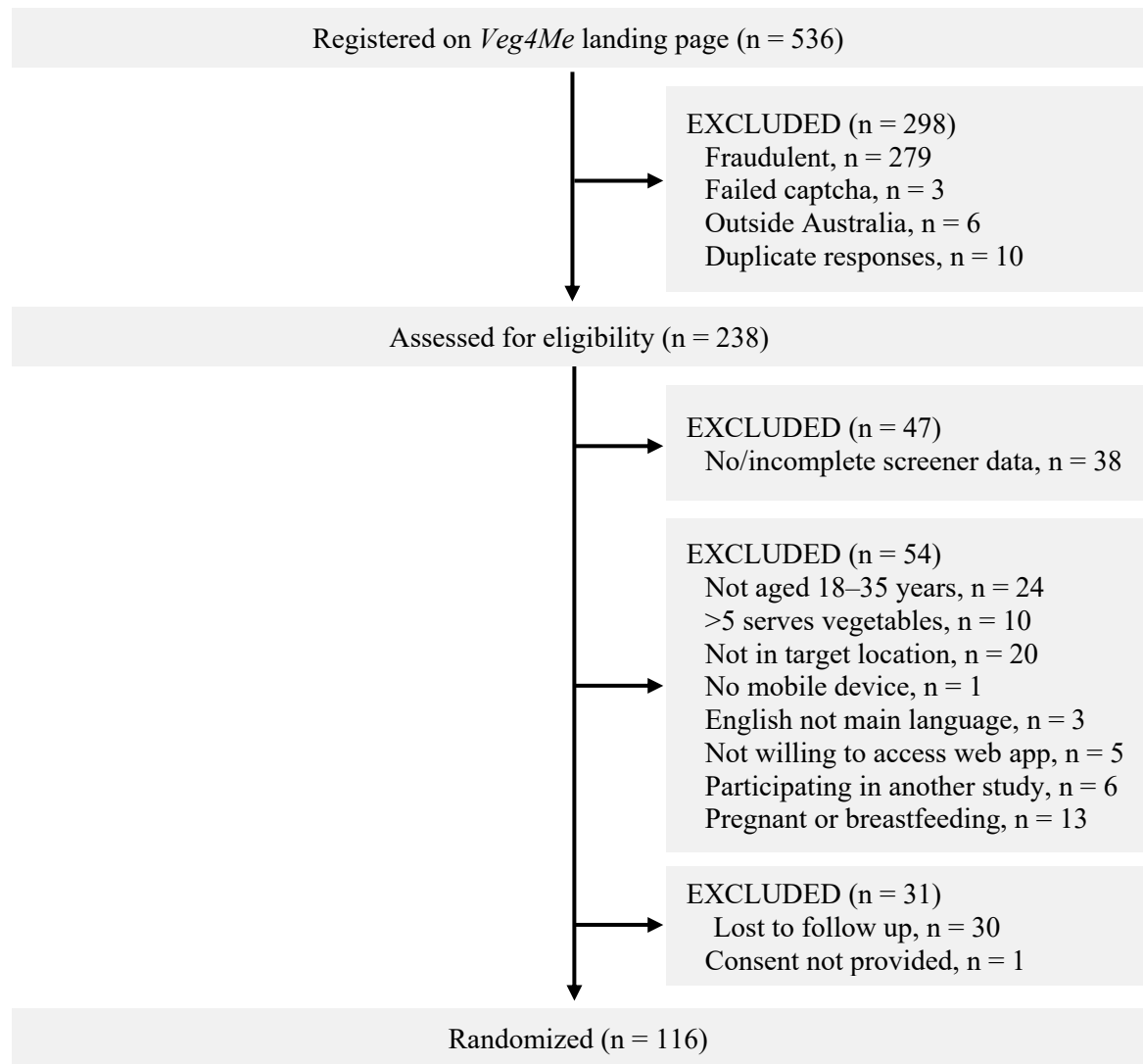

**Figure S1.** Participant flow diagram in the *Veg4Me* study.

Supplement: Supplementary file 1 [file nutrients-18-01646-s001.zip › Figure S1.pdf]
